# Sodium tanshinone IIA sulfonate inhibits tumor growth via miR-138 upregulation in intermittent hypoxia-induced xenograft mice

**DOI:** 10.18632/aging.205531

**Published:** 2024-02-08

**Authors:** Xiao-Bin Zhang, Qi-Feng Gan, Xiu-Zhen He, Ya-Ting Yuan, Ling-Wang, Mao-Hong Huang, Ping-Yang Hong

**Affiliations:** 1 Department of Pulmonary and Critical Care Medicine, Zhongshan Hospital of Xiamen University, School of Medicine, Xiamen University, Xiamen, Fujian, People's Republic of China; 2 The School of Clinical Medicine, Fujian Medical University, Fuzhou, Fujian, People’s Republic of China; 3 School of Medicine, Xiamen University, Xiamen, Fujian, People’s Republic of China

**Keywords:** sodium tanshinone IIA sulfonate, intermittent hypoxia, tumor, miR-138

## Abstract

Purpose: We studied the functions of sodium tanshinone IIA sulfonate (TSA) in inducing tumor growth in obstructive sleep apnea (OSA)-mimicking intermittent hypoxia (IH) xenograft mice and the underlying potential molecular mechanism.

Methods: RNA sequencing was conducted to screen the differentially expressed microRNAs in cell lines exposed to IH with or without TSA treatment. As part of the 5-week *in vivo* study, we treated xenograft mice with 8-h IH once daily. TSA and miR-138 inhibitors or mimics were administrated appropriately. In addition, we performed real-time quantitative polymerase chain reaction (RT-PCR), Western blotting, enzyme-linked immunosorbent assay (ELISA), immunohistochemistry (IHC), microvessel density (MVD), and terminal deoxynucleotidyl transferase dUTP nick-end labeling (TUNEL) assays.

Results: RNA sequencing and RT-PCR results demonstrated that TSA increased the levels of miR-138 under IH conditions *in vitro*. TSA reduced the IH-stimulated high levels of hypoxia-induced factor-1α and vascular endothelial growth factor. Furthermore, IH contributed to high tumor migration, invasion, MVD, and low apoptosis. TSA attenuated IH-mediated tumor proliferation, migration, invasion, MVD, and increased apoptosis, whereas miR-138 inhibitor interrupted the effect of TSA on treating IH-induced tumor behaviors.

Conclusions: OSA mimicking IH facilitates tumor growth and reduces miR-138 levels. TSA inhibits IH-induced tumor growth by upregulating the expression of miR-138.

## INTRODUCTION

Increasing evidence has demonstrated that obstructive sleep apnea (OSA) is independently linked to cancer incidence and mortality [1–4]. Our previous studies and those conducted by other researchers confirmed that intermittent hypoxia (IH), an OSA marker, promotes tumor growth and progression [5–7]. The potential molecular mechanism of IH aggravating tumor progression is still elusive, and it is speculated that IH-induced systemic inflammation, oxidative stress, and immune dysfunction could be involved.

As a non-coded RNA, increasing evidence has demonstrated that miR-138 protects against tumor growth [8, 9]. Sustained hypoxia contributes to reduced miR-138 levels, whereas upregulated miR-138 levels protect the cells against hypoxia-induced injury [10, 11]. IH is significantly different from sustained hypoxia and is closer to ischemia–reperfusion damage [12, 13]. The changes and relevant pathophysiological effects of miR-138 in IH or OSA subjects have poorly been studied.

Sodium tanshinone IIA sulfonate (TSA), extracted from the Chinese herbal medicine *Salvia miltiorrhiza*, is a natural compound with anti-oxidation and anti-inflammatory properties [14]. We have previously demonstrated that TSA can reverse the IH-induced tumor high oxidative stress and low apoptosis levels [6]. However, the exact mechanism remains unclear.

In the present study, we elucidated how IH affected miR-138 levels and explored the therapeutic functions of TSA in IH-induced mice.

## MATERIALS AND METHODS

### Chemicals and reagents

The Lewis lung cancer (LLC) cell lines were purchased from the Cell Center of Shanghai Institutes for Biological Sciences, China. The TSA injection was obtained from Shanghai No. 1 Biochemical and Pharmaceutical Co. Ltd. (Shanghai, China). TRIzol reagent was brought from Invitrogen, Carlsbad, CA, USA. Radioimmunoprecipitation assay (RIPA) lysis buffer was provided by Solarbio, Beijing, China. Bicinchoninic acid (BCA) protein assay kit was from Beyotime, Beijing, China. MiR-138 agomir and antagomir were provided by RiboBio Company (Guangzhou, China). SYBR Premix Ex Taq was from Takara, Dalian, China. Anti-hypoxia-induced factor-1α (HIF-1α), anti-B-cell lymphoma 2-associated protein X (Bax), and anti-Caspase-3 were provided by Abcam Company, Waltham, MA, USA. Anti-vascular endothelial growth factor (VEGF), anti-N-cadherin, anti-catenin, anti-matrix metalloproteinase 9 (MMP9), anti-matrix metalloproteinase 3 (MMP3), anti-CD34, and anti-β-actin antibodies were provided by Santa Cruz Biotechnology, Santa Cruz, CA, USA. The TUNEL assay kit was purchased from Roche Diagnostics Corporation, Shanghai, China. The C57BL/6J mice were acquired from the Laboratory Animal Center of Xiamen University.

### Cell lines

Cells were routinely cultivated in Dulbecco’s modified Eagle’s medium containing 10% fetal bovine serum and kept in a 5% CO_2_ incubator at 37° C under routine passage.

### IH exposure *in vitro* and TSA administration

We later categorized the cells as control (CTL), IH, and IH + TSA groups. The cells in IH and IH + TSA groups were incubated in an oxygen control incubator (Smartor 118, Guangzhou, China) with 1%, 1%–21%, 21%, and 21%–1% O_2_ for 5 min each during the 24-h period. Cells in the CTL group were cultured under a 5% CO_2_ atmosphere. The cells were co-incubated with TSA (10 μg/mL) [15] in the IH + TSA group.

### RNS sequencing and miR-138 differential expression analysis

The TRIzol reagent was used to extract the total RNA from LLC cells. RNA sequencing was completed in RiboBio company. Briefly, we used the total RNA (50 ng) to construct a library. Thereafter, library amplification and sequencing were completed with the HiSeqTM 2500 system using the HiSeq Rapid SBS Kit V2 and HiSeq Rapid SR Cluster Kit V2. Reads mapping to the miRNAs were counted using the high-throughput sequencing software. Differential expression (DE) of miRNAs was evaluated using the DE gene sequence (DEGseq) algorithm, and DEGseq software was used to obtain the statistically significant DE genes with thresholds of | log2 (fold_change) | >2 and adjusted *p*-value < 0.05.

### Animal groups and IH exposure

Animal experiments were conducted in line with the Guidelines for the Care and Use of Laboratory Animals [16]. Altogether, 30 male C57BL/6J mice were kept in standard cages under the 12-h/12-h light/dark cycles, with free access to food and water. According to the IH exposure, TSA administration, miR-138 agomir, and antagomir injection, the animals were randomized as CTL, IH, IH + TSA, IH + TSA + miR-138 mimic, and IH + miR-138 inhibitor groups. Under IH conditions, mice were placed in a chamber, with oxygen content altering from 21% to nadir 6%–8% within 120 s for an 8-h period every daytime for 5 weeks consecutively, as mentioned previously [6]. During the experiment, animals were kept in standard cages with the 12-h/12-h high-dark cycle, with free access to food and water. The body weights of mice were determined weekly.

### Tumor implantation, TSA intraperitoneal injection, and miR-138 agomir and antagomir intratumoral injection

At 1-week post-IH exposure, mice were subcutaneously injected with LLC cells (1 × 10^6^/100 μL of PBS) in the right flank. After the tumor grew to an appropriate volume (around 5–7 days post-LLC administration), TSA (10 mg/kg) was administered to the animals in IH + TSA and IH + TSA + miR-138 inhibitor groups daily through intraperitoneal injection [6]. Mice in the IH + TSA + miR-138 inhibitor group received miR-138 antagomir intratumoral injection (25 nmol diluted in 100 μL of PBS per mouse), whereas those in the IH + miR-138 mimic group received miR-138 agomir injection (25 nmol per mouse). The tumor length and width were measured per 3 days to calculate the tumor volume using the following formula: tumor volume = width × length/2.

### Serum and tumor tissue sample preparation

At the end of IH exposure (5 weeks), pentobarbital was injected to anesthetize the mice. Mice were administered cardiac puncture for exsanguination, and the serum was extracted for further analysis. The tumor was excised, weighed, either immersed in liquid before transfer onto the –80° C freezer or immersed in buffered 10% formalin for histological examination.

### Real-time quantitative polymerase chain reaction (RT-qPCR)

TRIzol reagent was used to extract RNA. The miR-138 expression was detected through RT-qPCR with SYBR Premix Ex Taq using the following conditions: 15 s at 95° C, 20 s at 60° C, and 25 s at 72° C for 40 cycles on an ABI 7500 (Applied Biosystems, Foster City, CA, USA). The following primers (5’-3’) were used: miR-138: AGCTGGTGTTGTGAATC (forward, F) and CAGTGCAGGGTCCGAGGTAT (reverse, R), U6: CTCGCTTCGGCAGCACA (F) and AACGCTTCACGAATTTGCGT (R). The 2^–ΔΔCT^ approach was used for data analysis.

### Enzyme-linked immunosorbent assay (ELISA)

We conducted an enzyme-linked immunosorbent assay (ELISA) to detect VEGF (pg/mL) concentration in the serum, following the instructions of the manufacturer (R&D Systems, Minneapolis, MN, USA).

### Western blotting

The tumor tissue was used to extract the proteins using the RIPA lysis buffer (Solarbio, Beijing, China) in a glass homogenizer on ice. The BCA assay kit (Beyotime, Beijing, China) was used to detect the total protein content. Protein aliquots were separated through 10% sodium dodecyl sulfate–polyacrylamide gel electrophoresis (SDS–PAGE) before being transferred into polyvinylidene difluoride membranes. After 1-h blocking using 5% defatted milk under ambient temperature, the membranes were subjected to overnight primary antibody incubation at 4° C: anti-HIF-1α (1:250), anti-VEGF (1:1000), anti-BAX (1:1000), anti-cleaved caspase-3 (1:1000), anti-N-cadherin (1:1000), anti-catenin (1:1000), anti-MMP9 (1:1000), and anti-MMP3 (1:1000) antibodies. After washing, the membranes were subjected to 1-h secondary antibody incubation under ambient temperature. Thereafter, the enhanced chemiluminescent (ECL) kit was applied to detect protein bands, with β-actin as the endogenous reference. ImageJ software (version 1.51) was used to quantify these protein levels.

### Hematoxylin–eosin staining and immunohistochemistry

The tumor tissue was embedded into the paraffin and was sliced into 5 μm slices for hematoxylin-eosin (HE) staining. Afterward, we performed immunohistochemistry (IHC) with an anti-HIF-1α antibody. The images were acquired and processed by a microscope. The Image-Pro Plus (version 6.0, Media Cybernetics, Rockville, MD, USA) was used to measure the integrated optical density (IOD).

### Microvessel density (MVD) and TUNEL assays

We determined the MVD using the IHC analysis using an anti-CD34 antibody and counted at 400× magnification [17]. The average MVD was obtained by averaging the values of the high-power fields from every group. In addition, we conducted the terminal deoxynucleotidyl transferase dUTP nick-end labeling (TUNEL) assay using a TUNEL staining kit according to the specific protocols. Both the numbers of apoptosis and total cells within the field of view (400× magnification) were counted, and the percentage was analyzed.

### Statistical analysis

The SPSS 22.0 software (IBM Corp., Armonk, NY, USA) and GraphPad Prism 5.0 (GraphPad Software, Inc., San Diego, CA, USA) were used for data analysis. Results are represented by means ±standard deviation and compared using the one-way analysis of variance and the post hoc test. *p* < 0.05 stood for statistical significance.

### Data and material availability

Data acquired or examined in the present work can be obtained in the published manuscript.

## RESULTS

### TSA improved reduced miR-138 levels induced by IH *in vitro*

**Figure 1:**
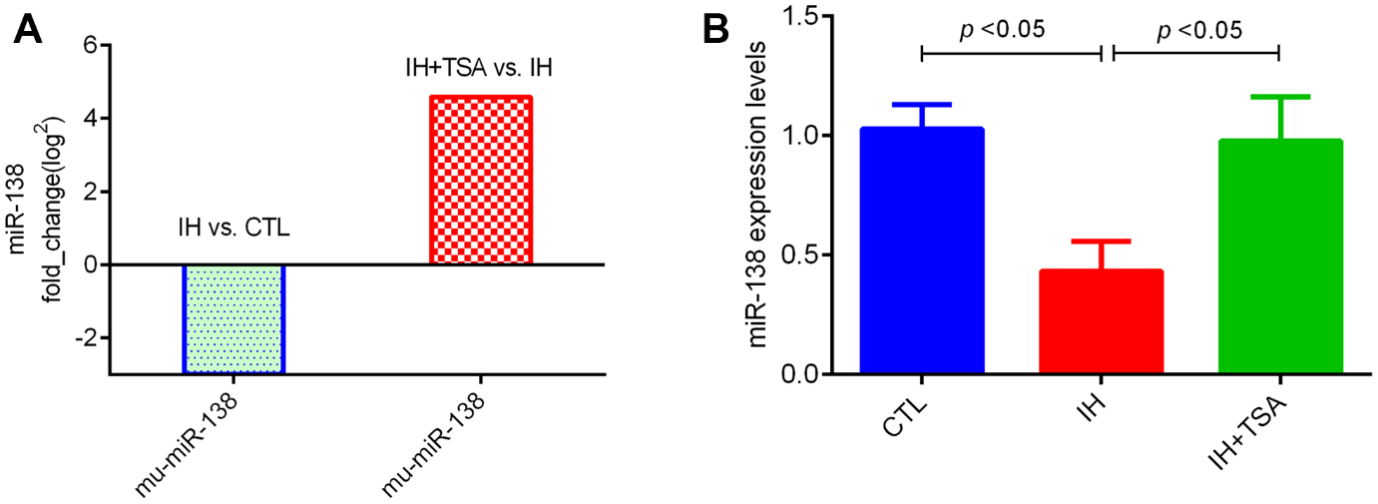
**Effect of IH and TSA on miR-138 expression *in vitro*.** (**A**) RNA sequence results demonstrate the fold_change in miR-138. (**B**) RT-qPCR verified the miR-138 expression in different groups.

The RNA sequencing results demonstrated that relative to the CTL group, the cells in the IH group exhibited low miR-138 fold_change. The administration of TSA increased miR-138 levels under IH conditions (Figure 1A). Subsequently, these results were confirmed by RT-qPCR (Figure 1B, both *p* < 0.05).

### TSA attenuated IH-induced tumor growth

**Figure 2:**
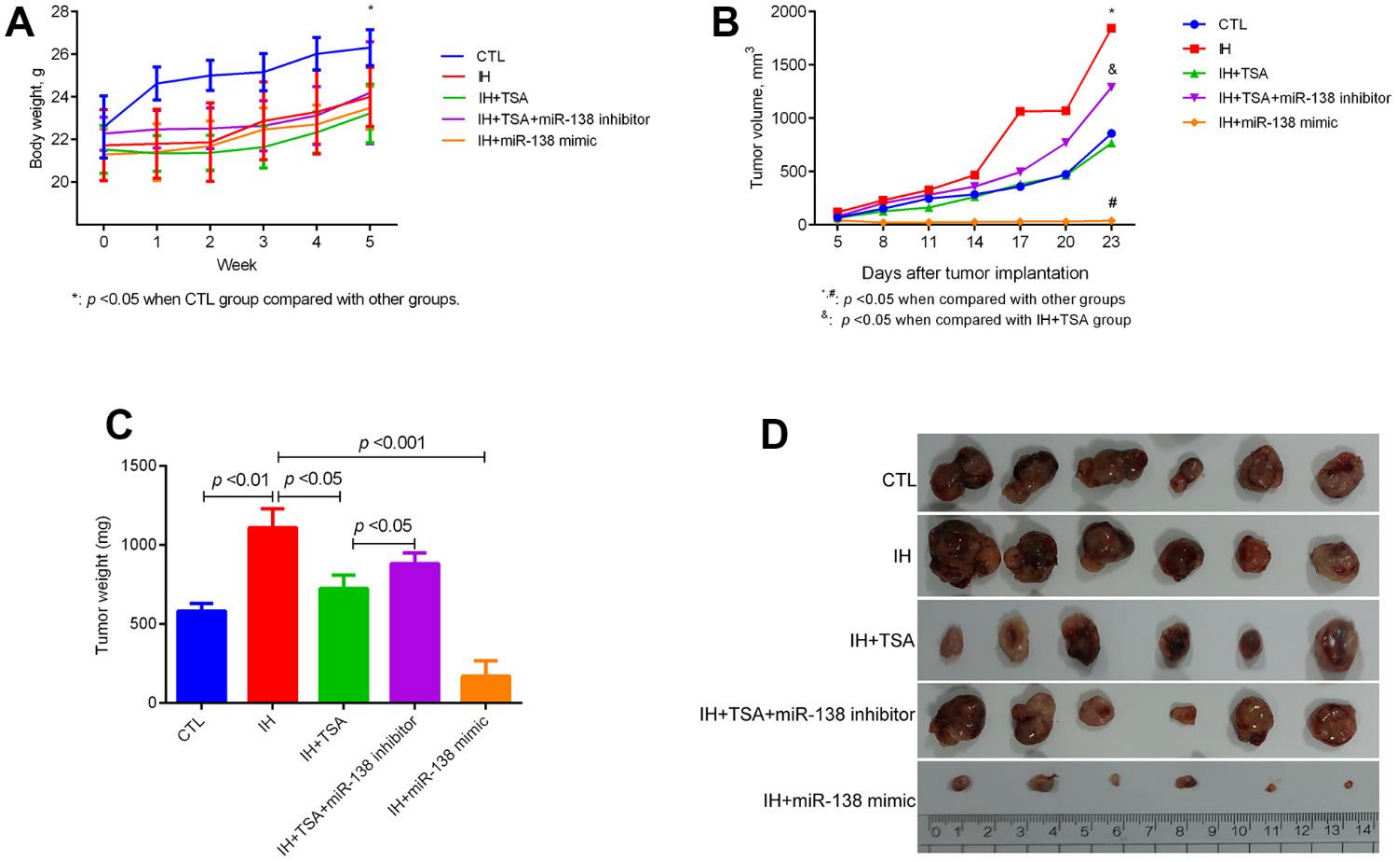
**Effect of TSA on tumor growth in IH-exposed xenograft mice.** (**A**) Change in body weight of mice at the indicated week. (**B**) Change in the tumor volume on the indicated day. (**C**) Comparison of tumor weight between different groups. (**D**) Tumor images in different groups.

The body weight of mice reduced following the IH exposure (Figure 2A, *p* < 0.05). The IH-induced increased tumor volume can be decreased by TSA or miR-138 mimic administration. Furthermore, an miR-138 inhibitor interrupted the therapeutic efficacy of TSA in tumor volume (Figure 2B, *p* < 0.05). Mice in the IH group displayed the highest tumor weight; TSA intraperitoneal injection and miR-138 mimic intratumoral injection significantly contributed to reduce tumor weight (*p* < 0.05 and *p* < 0.001). The therapeutic function of TSA was disturbed following the intratumoral injection of a miR-138 inhibitor (*p* < 0.05) (Figure 2C). The tumor images of each group are displayed in Figure 2D.

### TSA increased miR-138 expression in IH-exposed xenograft mice

**Figure 3:**
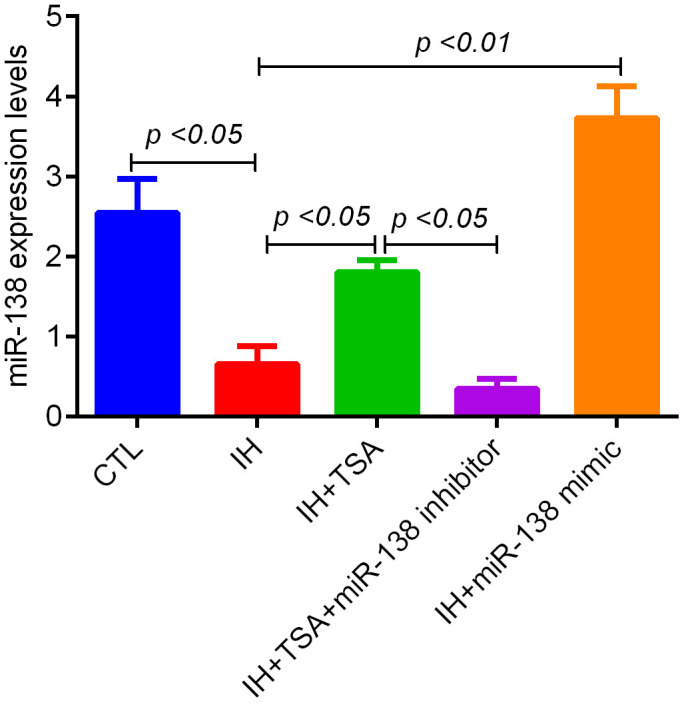
**Effect of TSA on miR-138 levels in IH-exposed xenograft mice.** RT-qPCR results demonstrate the miR-138 expression of tumor tissue between different groups.

The RT-qPCR results revealed that IH led to reduce miR-138 levels (*p* < 0.05 relative to the CTL group). The administration of TSA and miR-138 mimic increased the miR-138 levels following IH (*p* < 0.05 and 0.01, respectively). The TSA-induced increased miR-138 levels were inhibited by an miR-138 inhibitor (*p* < 0.05) (Figure 3).

### TSA decreased IH-induced HIF-1α and VEGF levels

**Figure 4:**
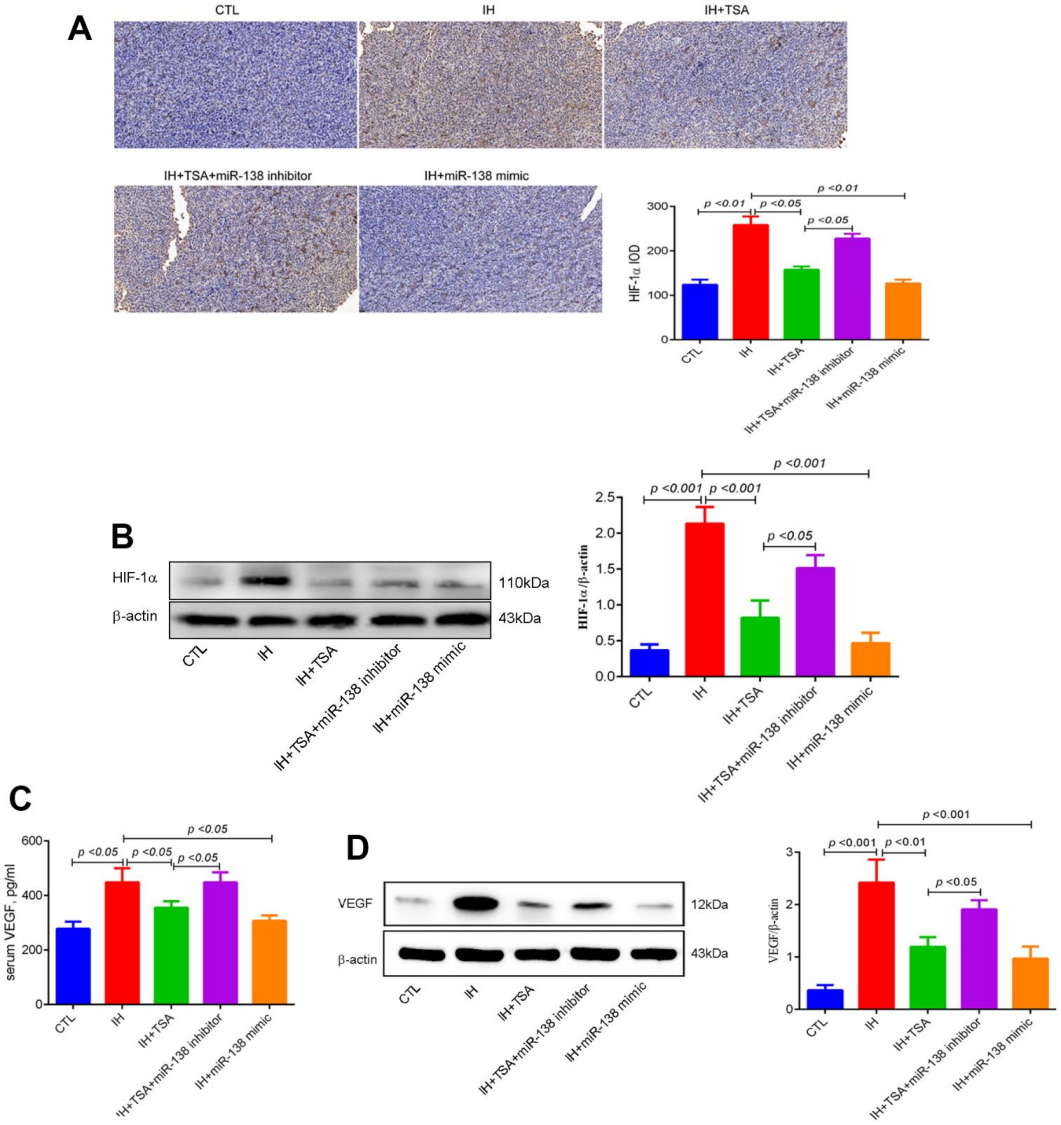
**Effects of TSA on HIF-1α and VEGF levels in IH-exposed xenograft mice.** (**A**) IHC of HIF-1α expression. (**B**) Western blotting results of HIF-1α. (**C**) Serum VEGF levels between groups with ELISA detection. (**D**) Western blotting results of VEGF.

IHC and Western blotting results demonstrated that IH-induced high HIF-1α levels. Under the IH condition, TSA and miR-138 mimic decreased HIF-1α levels (all *p* < 0.05), and the miR-138 inhibitor partly abolished the TSA function by increasing the levels of HIF-1α (*p* < 0.05) (Figure 4A, 4B). The expression of VEGF both in the serum and tumor tissue in the IH group increased (*p* < 0.05 and < 0.001). Similar to HIF-1α, the expression of VEGF decreased following the administration of TSA or miR-138 mimic under IH conditions. Subsequently, the reduced VEGF levels were partly upregulated by a miR-138 inhibitor (*p* < 0.05) (Figure 4C, 4D).

### TSA attenuated tumor migration, invasion, and MVD, and promoted apoptosis of IH-mediated xenograft mice

**Figure 5:**
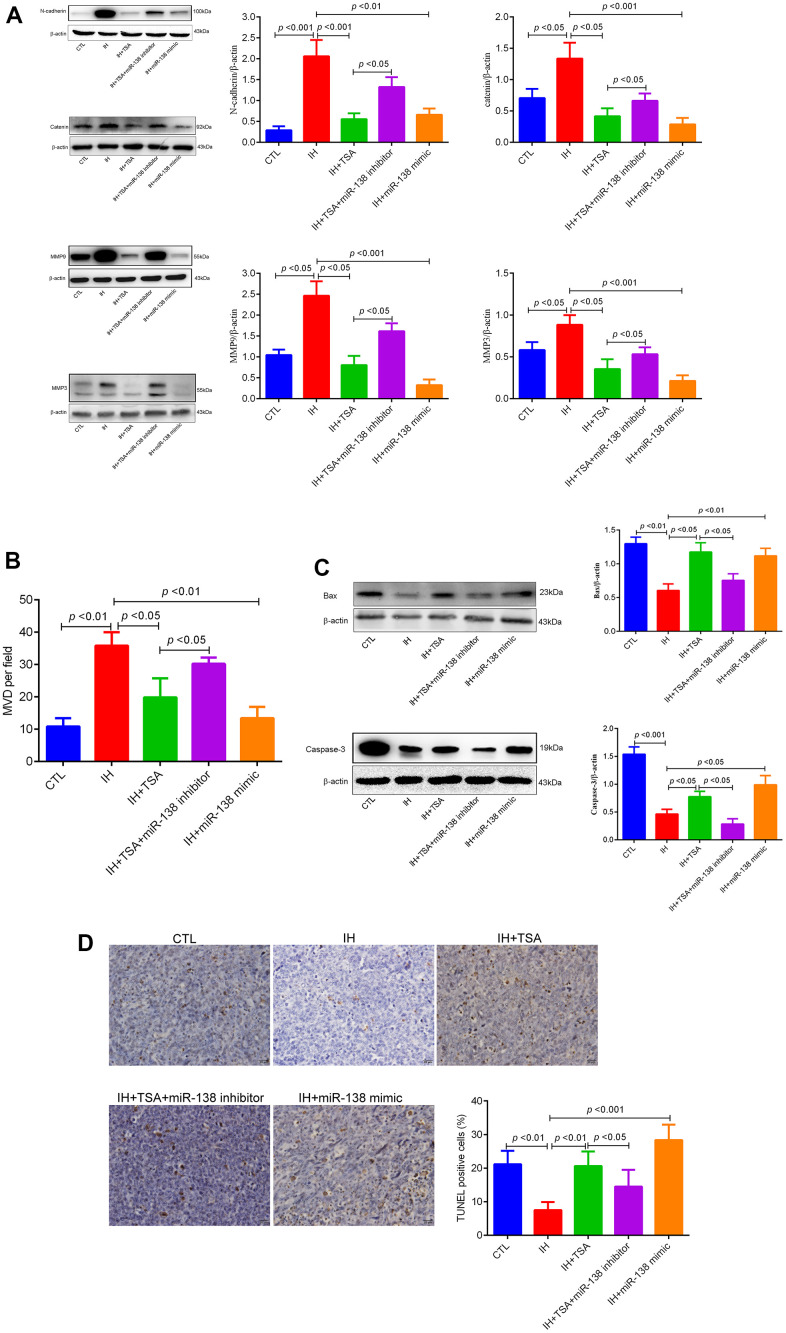
**Effects of TSA on tumor migration, invasion, MVD, and apoptosis in IH-exposed xenograft mice.** (**A**) Western blotting of tumor migration and invasion biomarkers, N-cadherin, catenin, MMP9, and MMP3. (**B**) MVD levels between different groups. (**C**) Western blotting of apoptosis biomarkers, BAX, and Caspase-3. (**D**) TUNEL assay images and results between different groups.

Western blotting results illustrated that the levels of N-cadherin, catenin, MMP9, and MMP3 increased in IH mice. Similar to the 138-mimic, TSA reduced the expression under IH conditions. The results demonstrated that TSA attenuated IH-induced tumor migration and invasion (Figure 5A). Figure 5B demonstrates that IH-induced high MVD levels were partly reversed by TSA or miR-138 mimic administration. IH contributed to low apoptosis levels, as evident from the reduced levels of BAX and Caspase-3, and TUNEL levels in the IH group; both TSA and miR-138 increased the apoptosis under IH condition (Figure 5C, 5D). The effect of TSA on tumor migration, invasion, MVD, and apoptosis was significantly reversed by miR-138 mimic under IH conditions, indicating that TSA attenuated tumor migration, invasion, MVD, and increased apoptosis via miR-138.

## DISCUSSION

In the present work, we demonstrated how TSA affected IH-induced xenograft mice and its potential molecular mechanism. IH promoted tumor development and reduced levels of miR-138. TSA inhibited IH-mediated tumor development and elevated miR-138 levels. The therapeutic function of TSA on tumors was reversed by an miR-138 inhibitor administration. The results indicated that TSA attenuated IH-induced tumor growth by stimulating the expression of miR-138.

Current evidence has demonstrated that OSA increases the incidence of cancer and mortality [1, 3, 18–21]. As a novel pathophysiological hallmark of OSA, IH has been demonstrated to contribute to tumor growth and progression in experimental studies [5, 7, 12, 17]. We have previously reported that IH leads to high levels of programmed death ligand 1 among mice with tumor [5]. The IH-induced xenograft mice displayed increased levels of MVD and VEGF [17]. Low apoptosis levels and high oxidative stress were observed among IH-exposed xenograft mice [6]. This study illustrated that IH enhanced tumor growth, accelerated tumor migration and invasion, promoted microvascular hyperplasia, and inhibited apoptosis. Although these studies provide certain clues, the definite mechanism of OSA-accelerated tumor growth remains elusive, and no effective therapeutic approach is available for OSA and cancer patients.

As the major lipophilic component of the Chinese Materia Medica *Salvia miltiorrhiza* Bunge, TSA exerts promising therapeutic effects on different disorders, including cancers, brain diseases, coronary heart disease, sepsis, and pulmonary diseases. Numerous experimental and clinical studies have demonstrated that TSA possesses effects against inflammation, apoptosis, and oxidation [14, 22]. The anti-tumor function of TSA has been demonstrated in several studies. For example, Zhang et al. [23] reported that TSA inhibited tumor development and enhanced the anti-tumor efficacy of anti-programmed death 1 antibody. As a therapeutic effect, TSA induced tumor cell apoptosis in colorectal cancer HCT116 cells [24]. The administration of TSA synergized with the inhibition of elongation factor-2 kinase that hindered A549 cell growth, invasion, and migration [25]. Our previous study [6] addressed the effects of TSA on tumor biological behaviors under the IH environment, these results demonstrated that TSA attenuated IH-mediated oxidative stress and promoted apoptosis of tumor cells. In the current study, we confirmed that TSA exerts anti-tumor effects by inhibiting tumor growth, migration, invasion, microvessel hyperplasia, and enhancing tumor apoptosis. The molecular mechanism underlying the therapeutic effect of TSA on tumor cells under IH conditions requires further studies.

Emerging studies [8, 26–29] have confirmed that miR-138 functions as a tumor suppressor by targeting several target genes associated with tumor proliferation, migration, invasion, and apoptosis. Under hypoxic conditions, miR-138 can serves as a tumor suppressor miRNA and is associated with hypoxia-induced factors [30]. For example, Zhang et al. [10] demonstrated that hypoxia-induced injury in cardiomyocytes resulted in reduced miR-138 levels. Emodin treatment can upregulate the levels and alleviate the hypoxia-induced injury. Gai et al. [11] reported that miR-138 remarkably reduced hypoxia/reoxygenation myocardial cell injury, whereas miR-138 mimics improved the injury. Although OSA-mimicked IH is familiar to hypoxia/reoxygenation or ischemia–reperfusion, rare study reported elevated miR-138 levels in tumor exposed to IH condition and subsequent treatment attempts. We first used the RNA sequence technology to detect that the fold_change in miR-138 levels reduced following IH exposure *in vitro*. Subsequently, we created OSA-mimicking IH xenograft mice and treated them with TSA and miR-138 mimic or inhibitor. The results demonstrated that IH exposure caused reduced miR-138 expression, promoted tumor proliferation, migration, and invasion, resulted in high microvessel density and low apoptosis levels. TSA increased the miR-138 levels under IH conditions. Similar to the miR-138 mimic, TSA treatment attenuated IH-induced tumor growth. The effect of TSA was interrupted by a miR-138 inhibitor. These results indicated that TSA inhibited IH-induced tumor progression by upregulating the expression of miR-138.

The current study had certain limitations. First, we did not address the exact molecular mechanism of how TSA mediates miR-138 expression and how miR-138 affects the tumor biological behavior under IH conditions. Second, each group comprised only six mice, and those under normoxic conditions did not receive TSA and miR-138 mimic and inhibitor treatment, which could probably influence the credibility of the results. Third, we only created an experimental animal model to address the function of TSA in IH-induced tumor development; *in vitro* and clinical studies are required to explore the possible molecular mechanism of TSA in OSA and tumor patients and verify its function.

## CONCLUSIONS

The levels of miR-138 declined among IH-induced tumor mice. TSA can attenuate IH-induced tumor growth via upregulating the expression of miR-138.

## References

[1] Kendzerska T, Povitz M, Leung RS, Boulos MI, McIsaac DI, Murray BJ, Bryson GL, Talarico R, Hilton JF, Malhotra A, Gershon AS. Obstructive Sleep Apnea and Incident Cancer: A Large Retrospective Multicenter Clinical Cohort Study. Cancer Epidemiol Biomarkers Prev. 2021; 30:295–304. 10.1158/1055-9965.EPI-20-0975 33268490 PMC7867627

[2] Kendzerska T, Gershon AS, Povitz M, Boulos MI, Murray BJ, McIsaac DI, Bryson GL, Talarico R, Hilton J, Malhotra A, Leung RS. Polysomnographic Markers of Obstructive Sleep Apnea Severity and Cancer-related Mortality: A Large Retrospective Multicenter Clinical Cohort Study. Ann Am Thorac Soc. 2022; 19:807–18. 10.1513/AnnalsATS.202106-738OC 34788198 PMC9116343

[3] Campos-Rodriguez F, Martinez-Garcia MA, Martinez M, Duran-Cantolla J, Peña Mde L, Masdeu MJ, Gonzalez M, Campo Fd, Gallego I, Marin JM, Barbe F, Montserrat JM, Farre R, and Spanish Sleep Network. Association between obstructive sleep apnea and cancer incidence in a large multicenter Spanish cohort. Am J Respir Crit Care Med. 2013; 187:99–105. 10.1164/rccm.201209-1671OC 23155146

[4] Tan BKJ, Teo YH, Tan NKW, Yap DWT, Sundar R, Lee CH, See A, Toh ST. Association of obstructive sleep apnea and nocturnal hypoxemia with all-cancer incidence and mortality: a systematic review and meta-analysis. J Clin Sleep Med. 2022; 18:1427–40. 10.5664/jcsm.9772 34755597 PMC9059590

[5] Huang MH, Zhang XB, Wang HL, Li LX, Zeng YM, Wang M, Zeng HQ. Intermittent hypoxia enhances the tumor programmed death ligand 1 expression in a mouse model of sleep apnea. Ann Transl Med. 2019; 7:97. 10.21037/atm.2019.01.44 31019947 PMC6462651

[6] Zhang XB, Chen XY, Sun P, Su XM, Zeng HQ, Zeng YM, Wang M, Luo X. Sodium Tanshinone IIA Sulfonate Attenuates Tumor Oxidative Stress and Promotes Apoptosis in an Intermittent Hypoxia Mouse Model. Technol Cancer Res Treat. 2020; 19:1533033820928073. 10.1177/1533033820928073 32431212 PMC7249596

[7] Hao S, Zhu X, Liu Z, Wu X, Li S, Jiang P, Jiang L. Chronic intermittent hypoxia promoted lung cancer stem cell-like properties via enhancing Bach1 expression. Respir Res. 2021; 22:58. 10.1186/s12931-021-01655-6 33596919 PMC7890965

[8] Zeng D, Xu H, Ji N, Li J, Zhou M, Dan H, Zhou Y, Zeng X, Jiang L, Chen Q. *In situ* measurement of miR-138 expression in oral squamous cell carcinoma tissue supports the role of this microRNA as a tumor suppressor. J Oral Pathol Med. 2019; 48:911–8. 10.1111/jop.12933 31323152

[9] Ou L, Wang D, Zhang H, Yu Q, Hua F. Decreased Expression of miR-138-5p by lncRNA H19 in Cervical Cancer Promotes Tumor Proliferation. Oncol Res. 2018; 26:401–10. 10.3727/096504017X15017209042610 28797320 PMC7844697

[10] Zhang X, Qin Q, Dai H, Cai S, Zhou C, Guan J. Emodin protects H9c2 cells from hypoxia-induced injury by up-regulating miR-138 expression. Braz J Med Biol Res. 2019; 52:e7994. 10.1590/1414-431X20187994 30810622 PMC6393853

[11] Gai YS, Ren YH, Gao Y, Liu HN. Astaxanthin protecting myocardial cells from hypoxia/reoxygenation injury by regulating miR-138/HIF-1α axis. Eur Rev Med Pharmacol Sci. 2020; 24:7722–31. 10.26355/eurrev_202007_22276 32744699

[12] Gozal D, Gileles-Hillel A, Cortese R, Li Y, Almendros I, Qiao Z, Khalyfa AA, Andrade J, Khalyfa A. Visceral White Adipose Tissue after Chronic Intermittent and Sustained Hypoxia in Mice. Am J Respir Cell Mol Biol. 2017; 56:477–87. 10.1165/rcmb.2016-0243OC 28107636

[13] Franczak A, Skomro R, Sawicka J, Bil-Lula I, Nocon A, Fenton M, Lawson J, Sawicki G. Serum matrix metalloproteinase-2 as a predictor of level of hypoxemia and severity of obstructive sleep apnea. Sleep Breath. 2021; 25:877–86. 10.1007/s11325-020-02200-3 33006024

[14] Guan R, Yao H, Li Z, Qian J, Yuan L, Cai Z, Ding M, Liu W, Xu J, Li Y, Sun D, Wang J, Lu W. Sodium Tanshinone IIA Sulfonate Attenuates Cigarette Smoke Extract-Induced Mitochondrial Dysfunction, Oxidative Stress, and Apoptosis in Alveolar Epithelial Cells by Enhancing SIRT1 Pathway. Toxicol Sci. 2021; 183:352–62. 10.1093/toxsci/kfab087 34515779

[15] Xu L, He D, Wu Y, Shen L, Wang Y, Xu Y. Tanshinone IIA inhibits cardiomyocyte apoptosis and rescues cardiac function during doxorubicin-induced cardiotoxicity by activating the DAXX/MEK/ERK1/2 pathway. Phytomedicine. 2022; 107:154471. 10.1016/j.phymed.2022.154471 36182795

[16] Albus U. Guide for the Care and Use of Laboratory Animals (8th edn). Laboratory Animals. 2012; 46:267–8. 10.1258/la.2012.150312

[17] Zhang XB, Yang YY, Zeng Y, Zeng HQ, Fu BB, Ko CY, Luo X, Du YP, Chen LD, Lai YT, Wu Y. Anti-tumor effect of endostatin in a sleep-apnea mouse model with tumor. Clin Transl Oncol. 2019; 21:572–81. 10.1007/s12094-018-1955-8 30293229

[18] Wu D, Zhao Z, Chen C, Lu G, Wang C, Gao S, Shen J, Liu J, He J, Liang W. Impact of obstructive sleep apnea on cancer risk: a systematic review and meta-analysis. Sleep Breath. 2023; 27:843–52. 10.1007/s11325-022-02695-y 36129602

[19] Cheong AJY, Tan BKJ, Teo YH, Tan NKW, Yap DWT, Sia CH, Ong TH, Leow LC, See A, Toh ST. Obstructive Sleep Apnea and Lung Cancer: A Systematic Review and Meta-Analysis. Ann Am Thorac Soc. 2022; 19:469–75. 10.1513/AnnalsATS.202108-960OC 34792438

[20] Yap DWT, Tan NKW, Tan BKJ, Teo YH, Tan VKM, See A, Toh ST. The Association of Obstructive Sleep Apnea With Breast Cancer Incidence and Mortality: A Systematic Review and Meta-analysis. J Breast Cancer. 2022; 25:149–63. 10.4048/jbc.2022.25.e11 35380020 PMC9250875

[21] Cheng H, Li D. Investigation into the association between obstructive sleep apnea and incidence of all-type cancers: a systematic review and meta-analysis. Sleep Med. 2021; 88:274–81. 10.1016/j.sleep.2021.05.031 34219029

[22] Zhou ZY, Zhao WR, Zhang J, Chen XL, Tang JY. Sodium tanshinone IIA sulfonate: A review of pharmacological activity and pharmacokinetics. Biomed Pharmacother. 2019; 118:109362. 10.1016/j.biopha.2019.109362 31545252

[23] Zhang R, Wang Y, Liu D, Luo Q, Du P, Zhang H, Wu W. Sodium Tanshinone IIA Sulfonate as a Potent IDO1/TDO2 Dual Inhibitor Enhances Anti-PD1 Therapy for Colorectal Cancer in Mice. Front Pharmacol. 2022; 13:870848. 10.3389/fphar.2022.870848 35571116 PMC9091350

[24] Zhou X, Pan Y, Wang Y, Wang B, Yan Y, Qu Y, Ke X. Tanshinones induce tumor cell apoptosis via directly targeting FHIT. Sci Rep. 2021; 11:12217. 10.1038/s41598-021-91708-z 34108553 PMC8190080

[25] Wang B, Gu X, Xiang BL, Zhao JQ, Zhang CH, Huang PD, Zhang ZH. eEF-2K knockdown synergizes with STS treatment to inhibit cell proliferation, migration, and invasion via the TG2/ERK pathway in A549 cells. J Biochem Mol Toxicol. 2022; 36:e23158. 10.1002/jbt.23158 35844142

[26] Zhang Q, Pan J, Xiong D, Zheng J, McPherson KN, Lee S, Huang M, Xu Y, Chen SH, Wang Y, Hildebrandt Ruiz L, You M. Aerosolized miR-138-5p and miR-200c targets PD-L1 for lung cancer prevention. Front Immunol. 2023; 14:1166951. 10.3389/fimmu.2023.1166951 37520581 PMC10372486

[27] Liu S, Dou L, Miao M, Man X, Wei B, Jiang Z, Ouyang Y, Ozaki T, Yu M, Zhu Y. HES1-mediated down-regulation of miR-138 sustains NOTCH1 activation and promotes proliferation and invasion in renal cell carcinoma. J Exp Clin Cancer Res. 2023; 42:72. 10.1186/s13046-023-02625-0 36973704 PMC10045948

[28] Wu J, Han X, Yang X, Li Y, Liang Y, Sun G, Wang R, Wang P, Xie S, Feng J, Sun H. MiR-138-5p suppresses the progression of lung cancer by targeting SNIP1. Thorac Cancer. 2023; 14:612–23. 10.1111/1759-7714.14791 36597175 PMC9968603

[29] Sha HH, Wang DD, Chen D, Liu SW, Wang Z, Yan DL, Dong SC, Feng JF. MiR-138: A promising therapeutic target for cancer. Tumour Biol. 2017; 39:1010428317697575. 10.1177/1010428317697575 28378633

[30] Sawai S, Wong PF, Ramasamy TS. Hypoxia-regulated microRNAs: the molecular drivers of tumor progression. Crit Rev Biochem Mol Biol. 2022; 57:351–76. 10.1080/10409238.2022.2088684 35900938

